# Compositional Analysis and Sustainable Valorization of the Calabrian Hazelnut cv. ‘Tonda Calabrese’ and Its Processing Derivatives

**DOI:** 10.3390/foods14183269

**Published:** 2025-09-20

**Authors:** Federica Turrini, Federica Grasso, Aseel Swaidan, Giosuè Costa, Sonia Bonacci, Antonio Procopio, Carmine Lupia, Raffaella Boggia, Stefano Alcaro

**Affiliations:** 1 Department of Pharmacy, University of Genova, Viale Cembrano 4, 16148 Genova, Italy; federica.turrini@unige.it (F.T.); federica.grasso@edu.unige.it (F.G.); aseel.swaidan@edu.unige.it (A.S.); 2 National Center for the Development of New Technologies in Agriculture (Agritech), 80121 Napoli, Italy; 3 Dipartimento di Scienze della Salute, Università “Magna Græcia” di Catanzaro, Campus “S. Venuta”, 88100 Catanzaro, Italy; s.bonacci@unicz.it (S.B.); procopio@unicz.it (A.P.); carmine.lupia@unicz.it (C.L.); alcaro@unicz.it (S.A.); 4 Net4Science Academic Spin-Off, Università “Magna Græcia” di Catanzaro, Campus “S. Venuta”, 88100 Catanzaro, Italy; 5 National Biodiversity Future Center (NBFC), 90133 Palermo, Italy; 6 Associazione CRISEA—Centro di Ricerca e Servizi Avanzati per l’Innovazione Rurale, Loc. Condoleo, 88055 Belcastro, CZ, Italy

**Keywords:** hazelnut cv. ‘Tonda Calabrese’, ultrasound-assisted extraction, enzyme-assisted extraction, sustainability, antioxidant activity, dietetics, nutraceutical, by-products valorization

## Abstract

Hazelnut cultivation is a strategic agricultural sector in Italy, with Calabria contributing through the native “Tonda Calabrese” cultivar, valued for its biodiversity. Despite its importance, data on the nutritional and compositional characteristics of this cultivar remain limited. In this study, hazelnuts from three different Calabrian producers were analyzed for morphological traits, proximate composition, and elemental content, using both conventional and non-destructive techniques such as CIELab color profiling and ATR-FTIR spectroscopy. The nuts showed high levels of essential micro-elements (Fe, Cu, Zn), aligning with previous findings on other cultivars, and showed no detectable pesticide residues, confirming their nutritional quality. Moreover, this study also aims to explore sustainable valorization strategies for hazelnut by-products, embracing circular economy principles in a “zero waste” approach, including oils and defatted flours. The extracted oils were evaluated for oxidative stability (peroxide value, p-anisidine, TOTOX index) and acidity, meeting Codex Alimentarius quality standards. The residual defatted flour was upcycled through eco-friendly methods, such as Ultrasound-Assisted Extraction (UAE) and Enzyme-Assisted Extraction (EAE), to isolate the polyphenol and protein fractions, respectively. Both extracts exhibited notable antioxidant activity (34.7–35.3 mmol Fe^2+^ eq/100 g and 64.3–82.2 mmol Fe^2+^ eq/100 g, respectively), suggesting their potential use as valuable ingredients for dietetic and nutraceutical applications.

## 1. Introduction

Hazelnuts (*Corylus avellana* L.) belong to the Corylaceae or Betulaceae family and are among the most widely consumed tree nuts globally [1,2]. Their origins date back to around 10,000 years ago, with fossil evidence of human consumption prior to cultivation in northern Europe. The Greeks and Romans were familiar with the plant, attributing to it symbolic and medicinal value. In Calabria, hazelnuts were already mentioned in historical works between the Middle Ages and the 17th century [3,4,5]. Cultivation in Cardinale (Catanzaro province, Calabria region, Italy) began in the late 18th century with plants introduced from Atripalda (Avellino) and was consolidated in 1850 with the first specialized orchard established by Carlo Filangieri. From there, hazelnut groves spread across the surrounding Serre Calabresi area [3].

Today, the municipality of Cardinale represents the main Calabrian production area, with about 500 hectares cultivated. The predominant ecotype, the ‘Tonda Calabrese’, was officially recognized as a regional biodiversity resource by the Italian Ministry of Agriculture in 2020 [6]. In Italy, hazelnut cultivation is concentrated in Campania, Lazio, Sicily, Piedmont, and Calabria, which ranks fifth with approximately 780 hectares [7]. On a global scale, Turkey dominates production (64%; corresponding to 765,000 tons), followed by Italy, Azerbaijan, the United States, Chile, and Georgia [8].

Hazelnuts are appreciated for their rich nutritional profile, including lipids (mainly oleic acid), dietary fiber, vitamin E, minerals, phytosterols (β-sitosterol), and antioxidant phenolics [9]. Several studies have documented significant variability in the nutritional composition of hazelnut cultivars [10,11,12,13]. Specifically, the moisture content is around 5–6%, protein content ranges from 14% to 23%, fat content from 46% to 64%, ash content from 2.3% to 3.5%, and carbohydrate content from 14% to 20%. Because environmental and genetic factors strongly influence the characteristics of plant-derived foods, consumers are increasingly demanding products with verifiable origin and authenticity, which underlines the importance of certification schemes such as PDO and PGI [14].

Finally, it should be noted that only about 10% of the global hazelnut production is consumed directly, while 90% is used in the food industry, especially as a flavoring in bakery, pastry, confectionery, and chocolate products. This has relevant economic and environmental implications due to by-product and waste generation [15].

In addition to hazelnut oils that have long been appreciated in numerous sectors (i.e., as gourmet oils and as ingredients in functional foods, food supplements, and cosmetics) [16], also the defatted flours obtained as their by-product can be valued as such and/or further exploited to obtain high-value extracts, as proposed in this research work.

**Figure 1:**
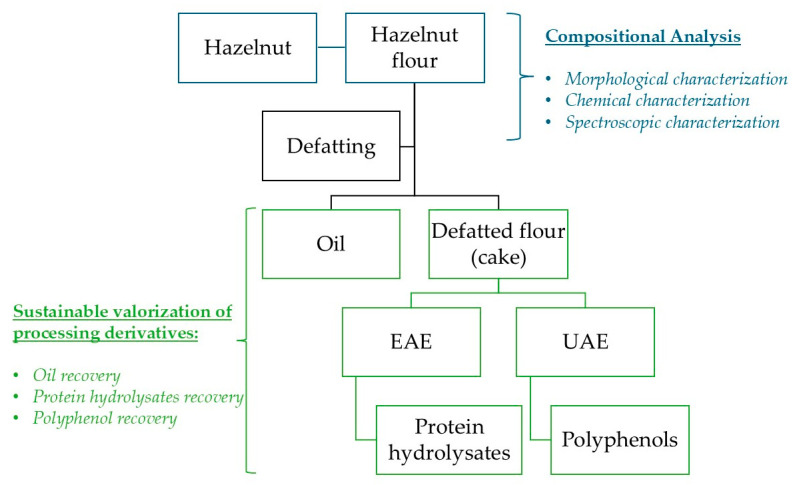
Graphical representation of the research.

This study aims to characterize a typical niche product—the Calabrian hazelnut from the municipality of Cardinale—and to explore the potential valorization of processing side-streams for the development of high-value products. Following the extraction of the lipid fraction, the focus shifted to the defatted solid residue (cake), which served as the starting material for the recovery of two bioactive fractions: polyphenols, extracted via Ultrasound-Assisted Extraction (UAE), and protein hydrolysates, obtained through Enzyme-Assisted Extraction (EAE) (Figure 1). Polyphenols and hydrolyzed peptides derived from agro-industrial residues are emerging as promising candidates for the formulation of high-value products across various sectors, including food, nutraceuticals, cosmetics, and pharmaceuticals [17,18]. These compounds exhibit a broad spectrum of potential beneficial properties, most notably antioxidant activity, which makes them particularly attractive for industrial applications aimed at developing innovative, sustainable, and health-promoting products.

## 2. Materials and Methods

### 2.1. Chemicals

All the chemicals and reagents were of analytical grade. Specifically, hexane, sulfuric acid, sodium hydroxide, ethanol 96%, ethyl acetate, methanol, DPPH• (1,1-diphenyl-2-picrylhydrazyl), Folin–Ciocalteu reagent, gallic acid, and Trolox (6-hydroxy-2,5,7,8-tertramethylchromane-2 carboxylic acid) were provided by Sigma-Aldrich Chemical Company (Steinheim, Germany). Boric acid (2%) with Sher indicator and catalyst tablets for the Kjeldahl analysis were purchased from Büchi Labortechnik AG (Flawil, Switzerland). The protease enzyme 3G PBN-66 L, a serine hydrolase obtained from the fermentation of *Bacillus licheniformis*, used at pH = 6 and 60 °C, was supplied by 3G SINCE 2000 (Barcelona, Spain). Regarding the Ferric Reducing Antioxidant Power (FRAP) test, Abcam© (Abcam, Caliph, ML, Cambridge, UK) provided the kit (ab234626). Nitric acid (HNO_3_, ≥69.0 TraceSELECT)) was purchased from Fluka analytical (Seelze, Germany). Purified water was obtained through a Milli-Q Integral 5 system (Millipore, Merck KGaA, Darmstadt, Germany). Two multielement ICP-MS calibration standards solutions (IMS-102, containing 10 μg mL^−1^ of As, Be, Cd, Co, Li, Ni, Se, Sr, and V, and IMS-103 containing 10 μg mL^−1^ of Sb and Sn) were purchased from Agilent (Santa Clara, CA, USA). Single-element analytical standards of Al, B, Ba, Ca, Cu, Fe, Mg, Mn, Mo, Na, P, and Zn containing 1000 μg mL^−1^ of each element were purchased from Ultra Scientific Italia (Bologna, Italy). Mercury standard solution 1000 μg mL^−1^ was purchased from Chem-Lab (Zedelgem, Belgium).

### 2.2. Samples

Representative samples were obtained from carefully selected batches of organically cultivated hazelnuts. In detail, samples of the seasoned *Tonda di Calabria* round variety hazelnut were collected by three different growers at the end of September 2023 in the Italian region of Calabria, specifically located in the Lombato and Signorello districts. Low-temperature dried (seasoned) hazelnuts were employed in this study, rather than fresh or roasted ones, to ensure sample stability and analytical reliability. Fresh hazelnuts were excluded due to their high perishability, while roasted samples were avoided as the variable degree of roasting could have led to confounding factors in the analytical determinations.

Samples were certified for organic cultivation, and commercial conformity was verified prior to analysis. The samples were vacuum-sealed and stored in the dark until analysis to prevent oxidative degradation. Each sample was labeled with the initials of the respective producer: RG, RR, and RGP.

### 2.3. Morphological Analysis

A set of 30 hazelnuts for each type of sample was randomly chosen to study the morphological traits, including the average weight of the nuts with and without the kernel using a technical balance with a sensitivity of 0.01 g. A vernier caliper was employed to measure their length (*A*), thickness (*B*), and width (*C*) to calculate their mean diameter (*Dp*), the index of roundness (*IR*), and the % of sphericity (*Ψ*), as indicated by the following equations [19,20]:

(1) Dp= (ABC)^1/3

(2) IR=(B+C)/(2A)  

(3) $$\psi =100\times \frac{Dp}{A}$$

### 2.4. Proximate Analysis

**Figure 2:**
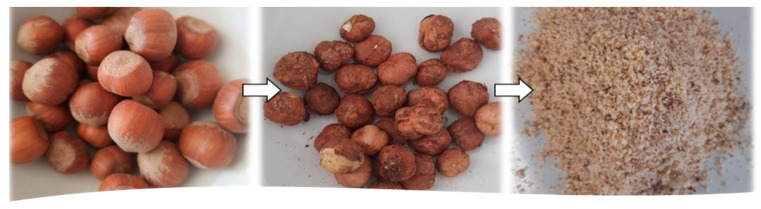
Shelling, grinding, and sieving processes.

The shells were removed, and nuts were blended in a mill (Grindomix 200M, Retsch, Haan, Germany) for 6 s at 5000 rpm and finally sieved to obtain a flour with uniform particle size of 500 μm (Figure 2).

The residual moisture was determined by applying the official procedure (AOAC 950.46B) and confirming it with a Sartorius thermogravimetric humidity analyzer (Massachusetts, USA). The protein content was assessed using the Kjeldahl method (AOAC 981.10), (N × 6.25), and also the determination of the ashes followed official methods (AOAC 942.05) [21]. The lipid fraction was extracted through a defatting suggested by Prodić et al. [22] with minor differences: the hazelnut flour was stirred three times with hexane 1/6 (*w*/*v*) for 30 min at room temperature and centrifuged at 5000× *g* for 10 min at 4 °C with a Refrigerated Benchtop Centrifuge 5810/5810 R (Eppendorf, Switzerland), then the supernatant was collected and the solvent was evaporated to isolate the lipid fraction, while the solid was recovered for further extractions of polyphenolic fraction (see Section 2.9) and enzymatic hydrolysates (see Section 2.10). Carbohydrates, including the fiber, were calculated by difference, i.e., the percentages of total protein, fat, and ash levels were subtracted from 100 to determine the total carbohydrate amount.

The pH of the hazelnut flour in water was measured at a ratio of 1:2.5 (*w*/*v*) at room temperature (25 ± 1 °C) with a pH meter (HI5221, Hanna Instruments, Villafranca Padovana, Padova, Italy) [23].

### 2.5. Determination of Mineral Content by ICP-OES and ICP-MS

In order to assess the mineral contents, elemental analysis of the hazelnuts was performed. Al, B, Ba, Ca, Cu, Fe, Mg, Mn, Mo, Na, P, and Zn contents were determined using an inductively coupled plasma optical emission spectrometer (ICP-OES Analyzer, iCAP 7400, Thermo Fisher Scientific Inc., Waltham, MA, USA) equipped with a glass concentric nebulizer coupled with a cyclonic spray chamber. The nebulizer gas flow and the auxiliary gas flow were set at 0.40 L min^−1^ and 0.50 L min^−1^, respectively. The coolant gas flow rate was adjusted to 12 L min^−1^. The ratio frequency (RF) power was set to 1150 W, and the peristaltic pump speed was maintained at 50 rpm.

Micro-element analysis (As, Be, Cd, Co, Hg, Li, Ni, Pb, Sb, Se, Sn, Sr, V) was carried out using an inductively coupled mass spectrometer (ICP-MS) (iCAP RQ, Thermo Fisher Scientific Inc., Bremen, Germany), operating with argon gas of spectral purity (99.9995%). The sample solutions were pumped by a peristaltic pump from tubes arranged on a CETAC ASX-520 auto-sampler (Thermo Scientific, Omaha, NE, USA). Instrument sensitivity, resolution, and mass calibration were optimized daily with the tuning solution (iCAP Q/RQ Tune aqueous multielement standard solution, Thermo Scientific Bremen, Germany), in order to maximize ion signals and to minimize interference effects due to high oxide levels, optimizing torch position, ion lenses, gas output, resolution axis, and background. The ICP-MS measurements were carried out under the following operating conditions: the RF power was set within a range of 500 to 1700 W, with reflected power maintained below 10 W. The plasma gas flow rate was set at 15 L min^−1^, the nebulizer gas flow at 1.00 L min^−1^, and the auxiliary gas flow at 0.80 L min^−1^. The instrument was operated using 5.00 mL min^−1^ helium (He) as the collision gas. The octopole bias (CCT bias) was set to −21 V, and the quadrupole (pole) bias to −18 V.

Prior to analysis, sample preparation was carried out using an Anton Paar Multiwave 5000 digestion system equipped with an XF100 rotor. In order to decontaminate PTFE vessels, a cleaning procedure was carried out by adding 4 mL of HNO_3_ and 4 mL of H_2_O in each vessel, in the following conditions: 1100 W for 15 min. After cleaning, vessels were rinsed with ultrapure water and dried as suggested by Chiesa et al. [24]. Aliquots of 0.2 g of each pooled sample were weighed directly into the PTFE vessel of the microwave system. The digestion was performed by adding 8 mL of HNO_3_. The operating conditions used for the microwave digestion were 800 W over 15 min, held at this power for 30 min. After digestion, samples were quantitatively transferred to a graduated polypropylene test tube and diluted with ultrapure water to 50 mL and stored at 4 °C until analysis. Each sample’s digestion was performed in triplicate. The analytical batch consisted of a set of calibration standards, samples, and a minimum of three procedural blanks. Each solution was measured in triplicate. All samples were analyzed in triplicate, and quantification was performed using a standard external calibration method. For each element, a minimum of six calibration points were used to construct the calibration curves. The concentration ranges were selected according to the expected elemental levels in the samples and the applied dilution factors. Specifically, the calibration intervals were as follows: 0.005–100 µg L^−1^ for trace elements; 0.002–1 mg L^−1^ for Ba, Cu, Mn, Mo, and Zn; 0.1–100 mg L^−1^ for Ca, Mg, Na, and K; and 0.01–10 mg L^−1^ for Al, B, Fe, and P. Calibration standards were prepared by appropriate dilution of stock solutions in 5% nitric acid (HNO_3_).

### 2.6. CIELab Color Analysis

The color analysis was carried out on samples of milled and sifted flours to obtain a sample with uniform grain size (500 microns). The 300–900 nm range was explored with a double-beam UV–Visible spectrophotometer (Agilent Cary 100 Varian Co., Santa Clara, CA, USA) combined with an integrating sphere (Varian DRA) at a resolution of 1 nm. A white Spectralon^®^ disk was used as a reference for the analyses. From the raw spectral data, the CIELab coordinates L* (lightness), a* (greenish–reddish), and b* (bluish–yellowish) were automatically extracted using the CIE D65 illuminant and the Cary100 Color program.

### 2.7. Attenuated Total Reflectance Fourier-Transform Infrared (ATR-FTIR) Analysis

ATR-FTIR spectroscopy was employed as a method of investigating the composition and structure of the hazelnut flours. After the recording of an initial background, spectra were acquired at room temperature from 4000 to 600 cm^−1^ at a resolution of 4 cm^−1^ using an FT-IR spectrophotometer (Perkin Elmer, Inc., Waltham, MA, USA), for a total of 8 scans per sample.

### 2.8. Evaluation of the Quality of the Lipid Fraction: Free Fatty Acids, Peroxide Value, and p-Anisidine Value

The quality parameters of the extracted hazelnut oil were assessed using the CDR FoodLab^®^ Fat system (CDR s.r.l., Florence, Italy), a compact analytical device based on photometric analysis. This system employs pre-vialed disposable reagents, which simplify sample handling and reduce exposure to hazardous chemicals, allowing for fast and safe determinations. The quality of the lipid fraction was evaluated in terms of acidity (free fatty acids, FFAs), primary oxidation products (Peroxide Value, PV), and secondary ones (p-Anisidine Value AV). The TOTOX index (TV), which provides an overall indication of both primary and secondary oxidation, was calculated as reported in the following equation [25].

TV  =  2PV  +  AV (4)

Each analysis was performed via spectrophotometric detection at specific wavelengths, following standardized protocols provided by the manufacturer. Briefly, the FFAs of the sample in the experimental conditions react with a chromogenic compound and decrease its color. The decreasing color, read at 630 nm, is proportional to the acid concentration of the sample, expressed as % of oleic acid. PV was determined based on the ability of peroxides (R-OO-R) to oxidize ferrous ions (Fe^2+^) into ferric ions (Fe^3+^). These ferric ions then form a colored complex, the intensity of which—measured at 505 nm—is directly proportional to the peroxide concentration in the sample. The results are expressed in milliequivalents of active oxygen per kilogram of oil (meq O_2_/kg). Secondary oxidation products react with p-anisidine, resulting in a measurable change in absorbance at 366 nm, which was used to calculate the AV. The results obtained are comparable in accuracy and reproducibility to those achieved using the official ISO/AOCS method [26], but with significantly reduced analysis time and simplified operational procedures.

### 2.9. Ultrasound-Assisted Extraction (UAE) of Polyphenols

The polyphenolic fraction was extracted from the defatted flour obtained after the treatment with hexane (see Section 2.4). The UAE was performed with a mixture of ethanol/water (70/30, *v*/*v*) in a ratio of 1:5 (*w*/*v*) using a Hielscher UP200St sonicator (Teltow, Germany) with a titanium sonotrode (14 mm of diameter), at a constant frequency of 26 kHz. The experimental conditions of sonication, previously optimized, were as follows: nominal power = 200 W, duty cycle = 80%, amplitude = 50%, and time = 30 min [27]. During the whole extraction, samples were maintained inside an ice bath that was replaced halfway to keep the temperature under 60 °C. Then, the solution was separated from the solid by centrifugation (Refrigerated Benchtop Centrifuge 5810/5810 R, Eppendorf, Hamburg, Germany) at 4 °C for 15 min at 7000× *g*; finally, the supernatant was filtered with filter paper (Whatman^®^ qualitative filter paper, Grade 1).

### 2.10. Enzymatic-Assisted Extraction of Hydrolysates

Enzymatic hydrolysis was performed on the defatted matrix obtained after the lipid extraction with hexane. Deionized water and defatted flour at a ratio of 1:12 (*w*/*v*) were included in a glass Pyrex flask and pH was measured to ensure it worked in the activity range of the enzyme (pH = 6–10). The selection of the enzyme was guided by evidence in the literature highlighting the effectiveness of alcalases in similar applications [28,29]. The proteolytic enzyme alcalase 3G PBN-66L (3G SINCE 2000, Barcelona, Spain) was added to the flask at 1% and the reaction was performed at 60 °C for 1 h in an incubator shaker (SKI 8R, Argo Lab, Kunshan, China). Afterwards, the enzyme was inactivated at 90 °C for 15 min and a centrifugation at 4 °C and 7000× *g* (Refrigerated Benchtop Centrifuge 5810/5810 R, Eppendorf, Hamburg, Germany) was carried out to recover the supernatant, which was filtered with filter paper under vacuum (Whatman^®^ qualitative filter paper, Grade 1).

### 2.11. Determination of Radical Scavenging Activity (RSA)

The RSA was determined by a DPPH• (1,1-diphenyl–2-picryl-hydrazyl) in vitro test by spectrophotometric analysis [30]. A calibration curve was prepared with Trolox as standard, measuring the absorbances at λ = 515 nm. The results were expressed as mg TEAC/100 mL of extract and/or mg TEAC/g sample (TEAC = Trolox Equivalent Antioxidant Capacity).

### 2.12. Determination of the Total Phenolic Compounds (TPC)

The Folin–Ciocalteu spectrophotometric method was employed to determine the TPC of the 3 samples of Calabrian hazelnuts, following the method reported by Turrini et al. [31], using an Agilent 8453 UV-Vis spectrophotometer (Waldbronn, Germany). The concentration of polyphenols was assessed using a calibration curve with gallic acid as standard (R^2^ = 0.9946). The TPC was measured as milligrams of gallic acid equivalent (GAE) extracted from 100 mL of extract (mg GAE/100 mL) and also expressed as mg GAE/g of defatted hazelnut flour.

### 2.13. Ferric Reducing Antioxidant Power (FRAP) Assay

The total antioxidant activity of the different extracts, i.e., the hydroalcoholic extracts and the hydrolysates, was measured using a commercial FRAP assay kit (Abcam^©^). The reduction of ferric iron (Fe^3+^) to ferrous iron (Fe^2+^), which results in a blue tint with a maximum absorption value at 594 nm, is used to measure antioxidant power. With an R^2^ value of 0.980, the ferrous ammonium sulfate standard curve was used to determine the sample solutions’ antioxidant capability. The results were expressed as Fe ^2+^ equivalents mM and mmol/g, and the sample solutions were made in EtOH 70% and water, with dilution 1:5 and 1:2 for the hydroalcoholic extracts and enzymatic hydrolysates, respectively.

### 2.14. Statistical Analysis

Results are presented as mean values ± standard deviation. All experiments were conducted with at least duplicate. Statistical significance was assessed using the Student’s *t*-test and One-way ANOVA followed by Tukey’s post-hoc test. Differences were considered statistically significant at *p* < 0.05.

## 3. Results and Discussion

### 3.1. Morphological Analysis

The distribution of weight was evaluated as a parameter indicative of the yield of shelled hazelnuts by performing a preliminary morphological analysis.

**Figure 3:**
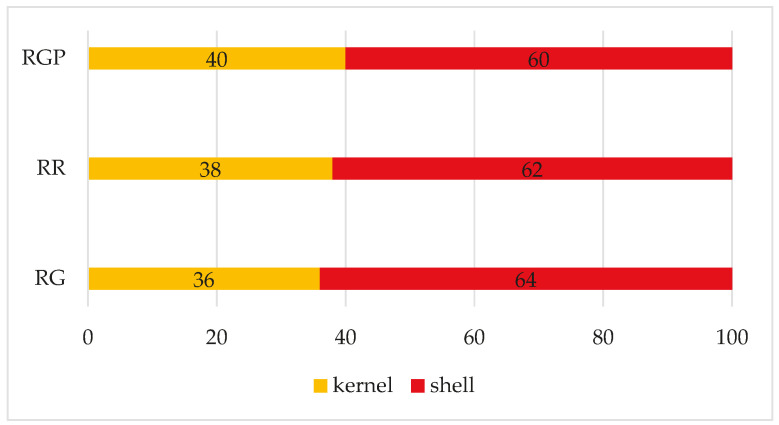
Distributions of kernel and shell.

According to the scientific literature, on average, distribution of weight is around 40–50% for most cultivars [32,33,34]. In the case of the three samples of round seasoned Calabrian hazelnuts, the yield of peeled ones is around 40%, and in some cases slightly lower, as highlighted in Figure 3. However, it should be emphasized that the shelling operation was carried out manually in the laboratory, unlike what usually happens in industry.

The average weight of hazelnuts with shells was evaluated and the results ranged from 2.8 ± 0.6 to 3.0 ± 0.8 g without highlighting statistically significant differences among the samples (One-way Anova *p*-value= 0.5366, F statistic= 0.627).

In addition, the morphological study of the parameters shown in Table 1 (Dp, mean diameter, IR, index of roundness, and Ψ, % of sphericity) was performed. Compared to IGP Piemonte commercial hazelnuts [35], the Calabrian hazelnuts, considered together with their kernels, were found to be slightly larger on average, but with a slightly lower weight if considered shelled, meaning that they have a thicker shell. Otherwise, the Calabrian hazelnuts have a greater roundness and sphericity in both the shelled and the unshelled version. From the scientific literature, it appears that among these parameters, sphericity is generally related to improved characteristics from the point of view of product crispness and a simpler subsequent roasting stage [36].

### 3.2. Proximate Analysis and pH

The proximate analysis reported in Table 2 was carried out on the hazelnut flour (Figure 2).

The humidity for the three types of hazelnuts was shown to oscillate between 5 and 6%, in accordance with the reference values for commercial hazelnuts, i.e., around 5% [12]. Lipids represent the major component, usually ranging between 50–70%. Italian cultivars contain around 60% of lipids, with differences that depend on several aspects, above all the cultivar, the geographical origin, and the climatic conditions. Notably, elevated temperatures are often associated with a reduction in oil content [37]. In the case of the samples analyzed, the lipids were extracted with organic solvents (hexane) and appear to constitute between 47 and 57% of the total composition. These differences may be related to slight variations in the harvest period, with RR collected later than RGP. Moderately high standard deviations observed in the fat contents during proximate analysis may be attributed to the presence of residual testa fragments. These components exhibit a distinct chemical profile—characterized by higher fiber and lower lipid content—which may have interfered with the accuracy of the measurements. Despite thorough sample preparation, the complete removal of skin residues in seasoned hazelnuts (without a roasting step) was not feasible, likely due to their strong adhesion and the structural characteristics of the skin matrix. Proteins can generally fluctuate between 10–24% [38], with an Italian average of around 15% [39], which corresponds to the values found for these samples by the Kjeldahl method. The ashes correspond to 2–3% of the total weight [40], as also verified by the samples analyzed. The fiber is usually around 11–14% [23], even if it is probable that in these samples there is a greater fiber content, which was estimated by difference to be between 20 and 30%.

The pH of hazelnuts is neutral and equal to 6.8 [41]; therefore, the data obtained are compliant, ranging from 6.58–6.72. pH is a critical parameter for product stability and shelf life, as a decrease may indicate fermentation or rancidity processes, none of which were observed in the examined samples, although this analysis will certainly need to be repeated and confirmed in subsequent years.

### 3.3. Quantification of Mineral Elements

The determination of macro-elements and trace minerals content in the hazelnuts was performed using external calibration curves and the results are reported below (Table 3).

Hazelnuts are known to be a good source of healthy beneficial minerals. In this study, the concentrations of the macro-nutrients Ca, Mg, Na, K, and P were measured. The highest concentrations of all considered macro-elements were found in the RR sample, except for sodium content (189 mg Kg^−1^). The Ca, Mg, and P concentrations, ranging from 967 to 1337 mg Kg^−1^, from 1544 to 2194 mg Kg^−1^, and from 26,544 to 3934 mg Kg^−1^, respectively, were comparable to the mean values discovered by previous authors [42,43,44]. Sodium content was approximately the same in all considered samples and ranged between 183 and 216 mg Kg^−1^. These values were half lower than the ones collected by Wojdyto et al. (540 mg Kg^−1^) [45], and much lower than those obtained by Tošić et al. (6616 mg Kg^−1^). Levels of potassium ranged from 6386 mg Kg^−1^ in the RG sample to 8551 mg Kg^−1^ in the RR sample. High potassium levels together with lower sodium content are important because they contribute to homeostasis maintenance. According to the detected contents of these elements, the Calabrian hazelnuts appear to be a good additional source of macro-nutrients in the human diet. The differences in macro-nutrients composition, compared to other studies, confirm how the geographical origin conditions the mineral contents.

Considering micro-elements, Calabrian hazelnut samples confirm their value as a good source of iron, copper, and zinc, essential minerals for overall health. In this context as well, the highest concentrations of these essential micro-elements were observed in the RR sample, where the levels of Fe, Cu, and Zn were 34.8 mg kg^−1^, 21.4 mg kg^−1^, and 23.7 mg kg^−1^, respectively. Overall, the micro-element profile of the Calabrian hazelnut samples aligns closely with data from earlier research on various hazelnut cultivars. For example, Simsek et al. analyzed multiple Turkish varieties and found notable differences in micro-element concentrations depending on cultivars. Their reported average levels ranged from 31.60 to 51.60 mg kg^−1^ for iron, 16.23 to 32.23 mg kg^−1^ for copper, and 22.03 to 44.03 mg kg^−1^ for zinc [46], in agreement with the results obtained. Likewise, Inaudi et al. [44] analyzed the inorganic component of the Italian ‘Tonda Gentile Romana’ hazelnut to authenticate its origin, reporting iron (38.8 mg kg^−1^), copper (13.3 mg kg^−1^), and zinc (18.0 mg kg^−1^) concentrations similar to those measured in these samples.

For the safety assessment of the hazelnut samples, the content of trace metallic trace elements (e.g., Cd, Ni, Pb, Cr) and the pesticide residue were evaluated (See Supplementary Materials). Notably, all samples were found to be free of pesticide residues.

### 3.4. CIELab Color and ATR-FTIR Analyses

Alongside traditional analyses, mainly carried out according to the official methods, the feasibility of using rapid innovative non-destructive techniques, such as color analysis through UV–Visible spectroscopy and ATR-FTIR analyses, was explored.

#### 3.4.1. CIELab Color Analysis

In addition to conventional analytical methods based on official protocols, this study also explored the potential application of innovative, rapid, and non-destructive approaches. Among these, colorimetric analysis plays a key role in the characterization of hazelnuts. Color analysis is generally used to define the origin of hazelnuts and to establish the correct degree of roasting, as it can be an index of the progress of the Maillard reaction (chemical browning) [47]. In Table 4, the CIELab parameters, i.e., L* (lightness), a* (redness/greenness), and b* (yellowness/blueness), obtained by UV–Visible spectroscopy equipped with an integrating sphere, were reported for the three hazelnut flours investigated.

While numerous studies have focused on the colorimetric attributes of hazelnuts post-roasting [36,48,49], variations in the color of raw hazelnuts may also be observed, potentially due to differences in geographical origin, harvest year, storage conditions, and cultivar. The obtained CIELab color data suggest that RR, harvested a little bit later, is darker (L* = 45.16) and less chromatically intense (a* = 2.26, b* = 11.62) than RG and RGP. Since no roasting occurred, these differences likely reflect natural ripening and compositional changes. The color profile of RR may also be influenced by its slightly higher lipid content compared to the other two samples, which could affect light absorption and scattering properties. In contrast, RGP, with higher lightness and lower chromatic values, likely represents less mature hazelnuts with a slightly different biochemical profile.

These findings demonstrate that CIELab color parameters can effectively reflect subtle compositional and maturity-related differences in raw hazelnuts, offering a non-destructive tool for preliminary quality assessment and cultivar differentiation. Moreover, the hazelnuts analyzed, compared to the color data reported in the literature [44], relating to Turkish hazelnuts, have a less intense color, defined by lower values of a* and b*, which means they have whiter pulp, and they appear to be brighter (higher L* values).

#### 3.4.2. ATR-FTIR Analysis

ATR-FTIR analysis, as a rapid non-destructive method, was chosen as the method to investigate the composition and the structure of the matrices under study. According to the literature it can be correlated with destructive proximate analysis and cultivar identification [50], due to its rapidity and high sensitivity, which allow for the detection of even subtle variations in sample structure and composition. Manfredi et al. [50] demonstrated the effectiveness of FTIR spectroscopy combined with chemometrics for fast classification of hazelnut cultivars from different origins, confirming its sensitivity and suitability for complex matrices such as hazelnuts. In this study, ATR-FTIR contributed to the comparative compositional analysis of hazelnuts from three different producers.

**Figure 4:**
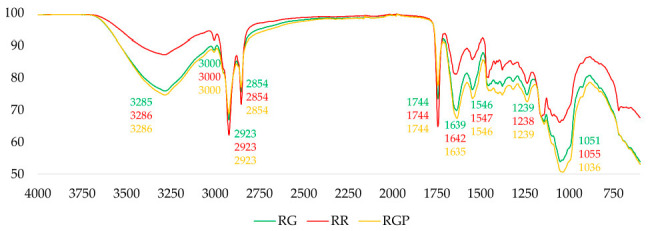
Spectra of the three hazelnut flours.

All three spectra (Figure 4) exhibited a broad absorption band around 3300 cm^−1^, corresponding to O–H and N–H stretching vibrations of proteins (Amide A) and polysaccharides [51]. The RG and RGP samples showed slightly broader and more intense peaks compared to RR, suggesting a higher content of proteins, phenolic compounds, and water [52] as residual moisture, parameters subject to change over time therefore potentially influenced by seasoning.

The C–H stretching vibrations of methylene and methyl groups around 2920–2850 cm^−1^ [53] were observed in all samples and may serve as an indicator of the degree of unsaturation in the acyl chains of phospholipids. RR displayed more pronounced peaks in this region, indicative of a higher lipid content, as confirmed by the destructive analysis.

A distinct absorption band near 1740 cm^−1^, representing carbonyl stretching, was present in all spectra, attributed to the C=O stretching of ester functional groups in triglycerides [52]. The RR sample exhibited higher intensity, suggesting a greater presence of intact lipid structures.

The amide I (C=O stretching) and amide II (N–H bending) bands (~1650 and ~1540 cm^−1^), characteristic of protein structures [54], were evident in all samples. RG and RGP showed marginally increased intensities and slight shifts in these bands, which may reflect protein denaturation or conformational changes due to seasoning.

The region 1200–900 cm^−1^, associated with C–O and C–C stretching vibrations in carbohydrates and polysaccharides [55], revealed complex and distinct patterns across the samples. RG and RGP demonstrated more pronounced and structured peaks, indicating possible alterations in carbohydrate composition or structure due to seasoning or thermal treatment.

Following the compositional analysis carried out to characterize the Calabrian hazelnut cultivar (Tonda Calabrese), we investigated the potential for sustainable valorization of its processing derivatives. These include both conventional by-products, such as hazelnut oil, and high-value fractions, such as polyphenol extracts and protein hydrolysates obtained from defatted flour (cake) (see Figure 4).

### 3.5. Analyses of the Lipid Fraction

#### 3.5.1. Evaluation of the Quality of the Lipid Fraction: Free Fatty Acids, Peroxide Value, and p-Anisidine Value

Among hazelnut by-products, oil represents a particularly valuable fraction. For the purposes of this study, hexane extraction was selected to facilitate comparison with the existing literature on other cultivars and due to its continued widespread use in industrial oil recovery processes.

To reduce the possibility of increasing the lipid oxidation, the extraction with hexane was performed at room temperature and the centrifugation was carried out at 4 °C, as detailed in Section 2.4. To assess the characteristics of the lipids, the acidity (FFAs) was analyzed as a parameter for the evaluation of total free fatty acids and for the degree of hydrolysis, along with the parameters of primary (peroxides, PV) and secondary oxidation (p-anisidine, AV). For all samples, values compliant with those established by the Codex Alimentarius for virgin vegetable oils [56] were obtained, as reported in Table 5 below.

#### 3.5.2. Antioxidant Power via DPPH

The antioxidant power of the hazelnut oils was evaluated via the DPPH spectroscopic test and the results are reported below (Table 6).

The results of the DPPH assay performed on oil extracted from the Tonda Calabrese hazelnut revealed good antioxidant activity, with % RSA values ranging from 22.2% (RG) to 29.2% (RGP) at a 1:10 dilution. These values are consistent with those reported in the literature for vegetable oils and phenolic extracts from hazelnuts [57]. The RGP sample, with a % RSA of 29.2 ± 0.17, exhibited the highest free radical scavenging capacity, suggesting a slightly higher concentration of bioactive compounds. A similar trend was observed in the hydrophilic polyphenolic fraction extracted using a hydroalcoholic solution (see Section 3.6), confirming the consistency between the different analyses.

### 3.6. Analyses on the Polyphenolic Fraction

The polyphenolic fraction was isolated starting from the defatted hazelnut flour using Ultrasound-Assisted Extraction (UAE) with a 70% ethanol hydroalcoholic mixture. The entire extraction process was conducted under controlled temperature conditions to ensure reproducibility and preserve the integrity of phenolic compounds. The results of the antioxidant potential analyses of the extracts are presented in Table 7 below.

Compared to the literature works of Kumar et al. 2016 [58] and Jakopic et al. 2011 [59], the values of the hydroalcoholic extracts obtained by UAE from the defatted flour of the hazelnuts examined were higher considering the content of total polyphenols, and as regards antioxidant activity they were comparable with similar extracts obtained by sonication [60]. The total polyphenol content (TPC) determined using the Folin–Ciocalteu spectrophotometric assay showed slight variations among producers, with values ranging from 1.94 to 2.29 mg GAE/g of defatted flour. The extract from the RGP sample exhibited the highest value, as already mentioned in the previous paragraph.

Regarding antioxidant activity, the results were consistent with those reported for similar extracts obtained using the same extraction technique. Specifically, the antioxidant capacity assessed by the FRAP method, which measures the ability of antioxidants to reduce Fe^3+^ to Fe^2+^, showed comparable values across the three extracts. The results of the FRAP assay were higher than the ones reported by Malgorzata et al. for hazelnuts, i.e., around 5 mmol/100 g [61], demonstrating the efficacy of the extraction method. Similarly, the DPPH assay, which evaluates the ability of antioxidants to donate an electron to the stable DPPH radical, confirmed good antioxidant capacity, ranging from approximately 76 to 93 mg Trolox equivalents (TEAC)/g of defatted hazelnut flour.

The RGP sample stood out for its higher values in both the Folin–Ciocalteu and DPPH assays, indicating a greater presence of phenolic compounds (both polar and non-polar) and enhanced antioxidant capacity. In contrast, the FRAP test revealed less pronounced differences among the samples.

A recent study demonstrated that Ultrasound-Assisted Extraction (UAE) applied to defatted hazelnut flour (press cake) [60] was the most effective among several tested methods, achieving a total radical scavenging activity of 2.88 mg TEAC/g of flour. However, this value is lower than those obtained in the present study. Conversely, when UAE was applied to the hazelnut perisperm (outer skin), a significantly higher radical scavenging activity was recorded, i.e., approximately 370 mg TE/g, confirming the high concentration of phenolic compounds in that specific fraction [62].

These findings suggest that, despite being derived from the endosperm, the polyphenolic fractions extracted from the Calabrian hazelnuts analyzed in this study exhibit particularly promising antioxidant potential, in some cases exceeding values reported in the literature for similar matrices.

### 3.7. Analyses of Hydrolysates

The hydrolysates were extracted using the innovative EAE starting from defatted flours (cake). Only preliminary analyses were carried out to assess the antioxidant power of these extracts using the FRAP test and the values are reported in Table 8.

The differences observed among the samples suggest variability in the composition of the protein fraction, potentially attributable to the presence of bioactive peptides or co-extracted phenolic residues. Further investigations on these fractions are currently in progress, and the preliminary analyses presented here serve as proof of concept, paving the way for promising future developments.

## 4. Conclusions

This study highlights the distinctive chemical and nutritional characteristics of the Tonda Calabrese hazelnut cultivar from Serre Vibonesi. The fruits showed consistent morphological traits, including high roundness and sphericity, which favor industrial processing, although they exhibited a lower shelling yield compared to more common varieties. Nutritionally, the hazelnuts contained 47–57% lipids and approximately 15% protein, with an ash content between 2–2.5%, supporting the cultivar’s favorable nutritional profile. These hazelnuts were also rich in essential trace elements, particularly iron, copper, and zinc. A near-neutral pH indicates good product stability and versatility in various food formulations. All samples were organically cultivated and free of pesticide residues.

Regarding the valorization of processing by-products, the lipid fraction demonstrated excellent oxidative stability, with low peroxide and p-anisidine values and a TOTOX index well below the thresholds for edible oils. Antioxidant activity reached up to 29.2% RSA, confirming the functional potential of the oil.

The defatted flour remaining after oil extraction proved to be a valuable source of bioactive compounds. Specifically, the polyphenolic fraction, extracted via Ultrasound-Assisted Extraction (UAE), showed high levels of total polyphenols (up to 2.29 mg GAE/g) and antioxidant capacity (up to 92.5 mg TEAC/g), exceeding values reported for comparable matrices. Protein extracts obtained through Enzyme-Assisted Extraction (EAE) also exhibited significant antioxidant activity (FRAP 64–82 mmol Fe^2+^/100 g), suggesting the presence of bioactive peptides and/or co-extracted phenolic compounds, which are currently under further investigation. Among the analyzed samples, RR—harvested slightly later than the others—displayed higher lipid and mineral content without signs of over-maturation. This was supported by favorable oxidative parameters and the highest FRAP values, indicating optimal ripeness and enhanced bioactivity of protein-derived compounds.

In conclusion, Tonda Calabrese hazelnuts and their processing derivatives represent promising sources of bioactive compounds, suitable for integration into high-value supply chains focused on sustainability, innovation, and local resource enhancement. Future research, along with the implementation of origin certifications such as PGI, could further strengthen the market positioning of this cultivar and support its application in nutraceutical and health-oriented products within a circular economy framework.

## Figures and Tables

**Table 1:** Mean diameter (Dp), roundness index (IR), and sphericity (Ψ) of hazelnuts with and without shell.

		Dp ^1^	IR ^1^	Tukey Test	Ψ ^1^
RG RR RGP	With shell	2.2 ± 0.4 ^a^ 2.3 ± 0.5 ^a^ 2.1 ± 0.4 ^a^	1.0 ± 0.1 ^a^ 1.0 ± 0.1 ^a^ 1.0 ± 0.1 ^a^	a a a	119 ± 18.9 ^a^ 119 ± 23.1 ^a^ 113 ± 16.5 ^a^
RG RR RGP	Without shell	0.9 ± 0.2 ^a^ 0.8 ± 0.2 ^b^ 0.8 ± 0.2 ^b^	0.9 ± 0.1^a^ 0.9 ± 0.1 ^a^ 1.0 ± 0.1 ^b^	a a b	62 ± 9.3 ^a^ 55 ± 9.3 ^b^ 60 ± 10.1 ^ab^

^1^ Results are reported as mean ± standard deviation (*n* = 30). Significant statistical differences for *p* < 0.05 are reported with different letters (a, b).

**Table 2:** Proximate analysis and pH.

g/100 g ^1^	RG	RR	RGP
Residual Moisture	6.0 ± 0.45 ^a^	5.0 ± 0.13 ^a^	5.6 ± 0.30 ^a^
Proteins	14.7 ± 0.37 ^a^	15.3 ± 0.42 ^a^	14.8 ± 0.06 ^a^
Lipids	52 ± 2.5 ^a^	57 ± 2.2 ^a^	47 ± 5.0 ^a^
Ashes	2.43 ± 0.02 ^a^	2.18 ± 0.08 ^b^	2.53 ± 0.08 ^a^
Carbohydrates (fiber included)	24.8	20.2	30.1
pH	6.72 ± 0.03 ^a^	6.58 ± 0.03 ^b^	6.71 ± 0.05 ^a^

^1^ Results are reported as mean ± standard deviation (*n* = 2). Significant statistical differences for *p* < 0.05 are reported with different letters (a, b).

**Table 3:** Macro- and micro-elements content in hazelnut samples.

^1^ mg Kg^−1^	RG	RR	RGP
Ca	998 ± 25.95	1337 ± 42.78	967 ± 19.28
Mg	1544 ± 32.42	2194 ± 50.46	1578 ± 27.31
Na	183 ± 5.49	189 ± 4.73	216 ± 7.01
K	6386 ± 74.11	8551 ± 96.53	6535 ± 56.23
P	2654 ± 17.16	3934 ± 33.27	2657 ± 20.76
Fe	26.02 ± 0.86	34.8 ± 1.01	26.4 ± 0.98
Cu	14.7 ± 0.35	21.4 ± 0.78	14.9 ± 0.56
Zn	16.46 ± 0.31	23.7 ± 0.98	16.42 ± 0.77

^1^ Results are expressed as mean ± standard deviation (mg Kg^−1^).

**Table 4:** CIELab color profiles of hazelnut flours.

	RG	RR	RGP
L* ^1^	44.68 ± 0.27 ^a^	45.16 ± 0.66 ^a^	57.70 ± 0.28 ^b^
a* ^1^	2.60 ± 0.08 ^a^	2.26 ± 0.25 ^a^	1.67 ± 0.14 ^b^
b* ^1^	13.20 ± 0.02 ^a^	11.62 ± 0.51 ^b^	12.40 ± 0.08 ^c^

^1^ Results are reported as mean ± standard deviation (*n* = 3). Significant statistical differences for *p* < 0.05 are reported with different letters (a–c).

**Table 5:** Monitoring of the acidity and oxidation of the oils.

	RG	RR	RGP	Limits Set by the Codex Alimentarius for Virgin Vegetable Oils
FFAs ^1^ (% Oleic Acid)	<1.0 ± 0.0	<1.0 ± 0.0	<1.0 ± 0.0	≤4.0 %
PV ^1^ (meq O_2_/kg)	<1.0 ± 0.0	<1.0 ± 0.0	<1.0 ± 0.0	≤15 meq O_2_/kg
AV ^1^	<0.5 ± 0.0	0.5 ± 0.0	<0.5 ± 0.0	-
TV ^1^	2.5	2.5	2.5	≤26

^1^ Results are reported as mean ± standard deviation (*n* = 2).

**Table 6:** Antioxidant power via DPPH of the oils.

	RG	RR	RGP
RSA % ^1^	22.20 ± 0.19	23.31 ± 0.03	29.20 ± 0.17
TEAC mg/100 mL ^1^	77.78 ± 0.82	82.57 ± 0.15	107.52 ± 0.73

^1^ Results are reported as mean ± standard deviation (*n* = 2).

**Table 7:** Antioxidant power of the hydroalcoholic extracts.

	RG	RR	RGP
^1^ FRAP (mM Fe^2+^ eq)	13.88 ± 0.19	13.90 ± 0.84	14.10 ± 0.31
FRAP (mmol Fe^2+^ eq/100 g defatted flour)	34.70 ± 0.46	34.75 ± 2.09	35.25 ± 0.77
Folin (mg GAE/100 mL)	39.2 ± 0.74	38.73 ± 0.01	46 ± 1.3
Folin (mg GAE/g defatted flour)	1.96 ± 0.04	1.94 ± 0.01	2.29 ± 0.06
^2^ DPPH (TEAC mg/100 mL)	1517 ± 21	1605 ± 17	1850 ± 9
DPPH (TEAC mg/g defatted flour)	75.8 ± 1.1	80.2 ± 0.8	92.5 ± 0.5

Results are reported as mean ± standard deviation (*n* = 2). ^1^ The positive control provided with the FRAP assay kit (Abcam, ab234626) was used according to the manufacturer’s instructions and yielded an Fe^2+^ equivalent concentration of 0.81 ± 0.07 mmol/L. ^2^ Ascorbic acid as a positive control was tested at multiple concentrations (0.1–1 mM) and this is reported in the Supplementary Materials.

**Table 8:** Antioxidant power of the hydrolysates.

	RG	RR	RGP
FRAP (mM Fe^2+^ eq) ^1^	26.80 ± 2.02	34.27 ± 0.38	31.67 ± 0.38
FRAP (mmol Fe^2+^ eq/100 g defatted flour) ^1^	64.33 ± 4.85	82.24 ± 0.91	76.02 ± 0.91

^1^ Results are reported as mean ± standard deviation (*n* = 2). The positive control provided with the FRAP assay kit (Abcam, ab234626) was used according to the manufacturer’s instructions and yielded an Fe^2+^ equivalent concentration of 0.81 ± 0.07 mmol/L.

## Data Availability

The original contributions presented in the study are included in the article/Supplementary Materials, further inquiries can be directed to the corresponding authors.

## Supplementary Materials

The following supporting information can be downloaded at: https://www.mdpi.com/article/10.3390/foods14183269/s1. Table S1. Trace minerals content in hazelnuts samples. Table S2. Gas chromatography tandem mass spectrometry (GC-MS) parameters of pesticides. Table S3. Liquid chromatography tandem mass spectrometry (LC-MS) parameters of pesticides. Figure S1: Ascorbic acid calibration curve (DPPH test).
