# Supplementary material for: Compositional Analysis and Sustainable Valorization of the Calabrian Hazelnut cv. ‘Tonda Calabrese’ and Its Processing Derivatives
Source: Foods. 2025 Sep 20;14(18):3269. doi: 10.3390/foods14183269 (PMC12470017; doi:10.3390/foods14183269)

Supplementary Materials

Compositional Analysis and Sustainable Valorization of the Calabrian Hazelnut cv. 'Tonda Calabrese' and Its Processing Derivatives

Federica Turrini [1,2,†], Federica Grasso [1,†], Aseel Swaidan [1], Giosuè Costa [3,4,*], Sonia Bonacci [3], Antonio Procopio [3], Carmine Lupia [3], Raffaella Boggia [1,5,*] and Stefano Alcaro [3,4,6]

[1] Department of Pharmacy, University of Genova, Viale Cembrano 4, 16148 Genova, Italy; federica.turrini@unige.it (F.T.); federica.grasso@edu.unige.it (F.G.); aseel.swaidan@edu.unige.it (A.S.)

[2] National Center for the Development of New Technologies in Agriculture (Agritech), 80121 Napoli, Italy

[3] Dipartimento di Scienze della Salute, Università "Magna Græcia" di Catanzaro, Campus "S. Venuta",
88100 Catanzaro, Italy; s.bonacci@unicz.it (S.B.); procopio@unicz.it (A.P.); carmine.lupia@unicz.it (C.L.); alcaro@unicz.it (S.A.)

[4] Net4Science Academic Spin-Off, Università "Magna Græcia" di Catanzaro, Campus "S. Venuta",
88100, Catanzaro, Italy

[5] National Biodiversity Future Center (NBFC), 90133 Palermo, Italy

[6] Associazione CRISEA—Centro di Ricerca e Servizi Avanzati per l'Innovazione Rurale, Loc. Condoleo, 88055 Belcastro (CZ), Italy

* Correspondence: gcosta@unicz.it (G.C.); raffaella.boggia@unige.it (R.B.)

† These authors contributed equally to this work.

Table S1. Trace minerals content in hazelnut samples. page 2

1. Analysis of pesticide residues page 2

1.1 QuEChERS Extraction Procedure page 2

GC-MS analysis page 3

Table S2. Gas chromatography tandem mass spectrometry (GC-MS) parameters of pesticides. page 3

1.3 LC-MS analysis page 6

Table S3. Liquid chromatography tandem mass spectrometry (LC-MS) parameters of pesticides. page 6

Table S1. Trace minerals content in hazelnut samples.

mg Kg^{-1}	RG	RR	RGP
**Al**	1.3 ± 0.016	2.61 ± 0.036	1.18 ± 0.009
**As**	0.012 ± 0.0001	0.009 ± 0.0001	0.015 ± 0.0001
**B**	24.3 ± 0.87	32.3 ± 0.39	24.91 ± 0.56
**Ba**	7.7 ± 0.18	18.8 ± 0.14	10.42 ± 0.32
**Cd**	0.0045 ± 0.0004	0.0083 ± 0.0001	0.0067 ± 0.0002
**Co**	0.17 ± 0.009	0.18 ± 0.004	0.12 ± 0.003
**Cr**	0.08 ± 0.0042	0.02 ± 0.0002	0.02 ± 0.0001
**Cu**	14.7 ± 0.35	21.4 ± 0.78	14.9 ± 0.56
**Fe**	26.02 ± 0.86	34.8 ± 1.01	26.4 ± 0.98
**Mn**	34.8 ± 1.11	80.32 ± 0.76	35.8 ± 1.21
**Mo**	0.09 ± 0.0021	0.06 ± 0.0012	0.05 ± 0.0012
**Ni**	0.26 ± 0.006	0.51 ± 0.006	0.41 ±0.003
**Pb**	0.04 ± 0.0004	0.05 ± 0.0003	0.04 ± 0.0002
**Sn**	0.04 ± 0.0001	0.07 ± 0.0002	0.05 ± 0.0001
**Zn**	16.46 ± 0.31	23.7 ± 0 .98	16.42 0.77

1. Analysis of pesticide residues

1.1 QuEChERS Extraction Procedure

Hazelnut samples were first homogenized, and 5 g of the homogenized material was placed into a centrifuge tube. QuEChERS extraction salts, specifically, 2 g of magnesium sulfate and 1 g of sodium acetate, were added to the sample. Subsequently, 5 mL of water, 5 mL of acetonitrile, and 100 µL of formic acid were added to the mixture. The tube was vortexed for 3 minutes and centrifuged at 5000 rpm for 10 minutes. After centrifugation, the upper organic layer was carefully collected and evaporated to dryness under a nitrogen stream. The resulting dry residue was reconstituted with 1000 µL of acetonitrile. This solution was transferred to a microcentrifuge tube containing 50 mg of PSA (primary secondary amine) and 200 mg of magnesium sulfate for clean-up. The mixture was then centrifuged at 4000 rpm for 5 minutes. Finally, the supernatant was filtered through a 0.45 µm PTFE syringe filter, and the extract was stored at -20°C until analysis. Both GC-MS and LC-MS methods were employed for the analyses.

1.2 GC-MS analysis

GC-MS/MS analysis was performed using an Agilent 7890B gas chromatograph coupled to an Agilent 7693A autosampler and an Agilent 7000D triple quadrupole mass spectrometer (GC/TQ). The system was equipped with a Multi-Mode Inlet (MMI) operated in hot splitless mode. The inlet temperature was initially set at 105 °C, then ramped at 600 °C/min to the final temperature of 300°C. The injection was performed in splitless mode with a splitless time of 3 min, and a sample volume of 2 µL was injected. Chromatographic separation was achieved using a VF-Xms capillary column (CP8809, 60 m × 0.25 mm i.d. × 0.25 µm film thickness). Helium was used as the carrier gas in constant flow mode at 1.9 mL/min. The oven temperature program was as follows: initial temperature 60 °C held for 3 min, ramped at 25 °C/min to 150 °C, then ramped at 3.5 °C/min to 300 °C and held for 10 min. Mass spectrometric detection was performed in electron ionization (EI) mode at 70 eV, and data were acquired in MRM mode.

Table S2. Gas chromatography tandem mass spectrometry (GC-MS) parameters of pesticides.

Name	Retention time (min)	Quantifying ions (*m*/*z*)	Qualifying ions (*m*/*z*)
2,4,5-trichlorophenol	14.86	198.0 -> 97.0	198.0 -> 135.0
2,4'-DDD	36.24	235.0 -> 165.2	235.0 -> 199.0
2,4'-DDE	33.86	246.0 -> 176.2	316.0 -> 246.0
2,4'-DDT	38.34	235.0 -> 165.2	235.0 -> 199.0
3,4-dichloroaniline	16.28	161.0 -> 90.1	161.0 -> 99.0
4,4'-DDD	38.34	235.0 -> 165.2	235.0 -> 199.0
4,4'-DDE	35.69	246.0 -> 176.0	315.8 -> 246.0
4,4'-DDT	40.25	235.0 -> 165.2	235.0 -> 199.0
4,4'-dichlorobenzophenone	31.56	250.0 -> 139.0	250.0 -> 215.0
Acetochlor	26.92	174.0 -> 146.0	223.0 -> 147.0
Acibenzolar-S-methyl	29.60	182.0 -> 181.0	135.0 -> 63.0
Aclonifen	39.07	212.0 -> 182.0	264.0 -> 194.0
Aldrin	30.37	263.0 -> 193.0	263.0 -> 228.0
alfa-BHC	22.97	216.9 -> 181.0	181.0 -> 145.0
Allethrin	32.53	273.9 -> 125.0	246.0 -> 121.0
Benfluralin	20.23	292.0 -> 264.0	292.0 -> 160.0
beta-BHC	25.08	216.9 -> 181.0	181.0 -> 145.0
Bifenox	43.26	189.1 -> 126.0	311.0 -> 279.0
Bifenthrin	41.61	181.0 -> 166.0	181.0 -> 153.0
Biphenyl	14.90	154.0 -> 152.0	154.0 -> 102.0
Boscalid	51.55	140.0 -> 76.0	342.0 -> 140.0
Bromacil	30.18	207.0 -> 190.0	205.0 -> 188.0
Bromadiolone	30.17	258.0 -> 178.0	258.0 -> 258.0
Bromophos-ethyl	33.20	359.0 -> 303.0	303.0 -> 285.0
Bromophos-methyl	31.25	328.9 -> 313.9	330.9 -> 285.9
Bromopropylate	42.68	341.0 -> 185.0	185.0 -> 157.0
Butralin	30.33	266.0 -> 220.0	266.0 -> 190.0
Captan+Captafol	17.77	151.0 -> 79.1	79.0 -> 51.0
Carbophenothion	44.87	153.0 -> 96.9	199.0 -> 143.0
Chlordane-cis	34.62	372.8 -> 265.8	373.0 -> 301.0
Chlordane-trans	34.01	372.8 -> 265.8	373.0 -> 301.0
Chlorfenson	35.73	175.0 -> 111.0	302.0 -> 175.0

Chlorobenzilate	37.34	251.1 -> 139.1	139.1 -> 111.0
Chlorpyrifos	29.60	199.0 -> 171.0	314.0 -> 258.0
Chlorsulfuron	24.08	175.0 -> 111.0	191.0 -> 127.0
Chlorthal-dimethyl	30.00	299.0 -> 221.0	300.9 -> 222.9
Clopyralid	34.94	112.1 -> 76.0	147.0 -> 112.0
Clorfenapyr	36.11	136.9 -> 102.0	247.0 -> 227.0
Cloropropylate	37.10	251.0 -> 139.0	139.0 -> 111.0
Cyfluthrin	49.42	163.0 -> 91.0	163.0 -> 127.0
Cyprodinil	32.16	225.2 -> 224.3	224.2 -> 208.2
Delta-BHC	27.18	216.9 -> 181.0	181.0 -> 145.0
Deltamethrin	44.56	181.0 -> 152.1	250.7 -> 172.0
Desethylterbuthylazine	21.94	201.0 -> 186.0	201.0 -> 68.0
Desmetryn	27.40	213.0 -> 58.1	198.0 -> 108.0
Dialifos	44.53	208.0 -> 181.0	208.0 -> 102.0
Dichlobenil	14.23	171.0 -> 100.0	171.0 -> 136.0
Dichlofenthion	26.67	278.9 -> 222.9	222.9 -> 204.9
Dichlofluanid	30.31	123.0 -> 77.0	167.0 -> 124.0
Dicloran	24.24	206.1 -> 176.0	208.0 -> 178.0
Dicofol	31.57	139.0 -> 111.0	252.0 -> 141.0
Dieldrin	36.39	262.9 -> 193.0	277.0 -> 241.0
Dimepiperate	33.24	119.0 -> 91.0	145.0 -> 112.0
Dinitramine	24.82	305.0 -> 244.0	307.0 -> 216.0
Dioxathion	24.20	125.0 -> 97.0	152.9 -> 96.9
Dipropetryn	29.52	255.1 -> 222.1	255.1 -> 180.1
Disulfoton	37.44	153.0 -> 97.0	153.0 -> 125.0
Disulfoton sulfone	34.41	213.0 -> 96.9	213.0 -> 153.0
Disulfoton sulfoxide	33.20	125.0 -> 96.9	97.0 -> 65.0
Ditalimfos	34.85	130.0 -> 102.1	148.0 -> 130.1
Edifenphos	40.07	173.0 -> 65.1	310.0 -> 109.1
Endosulfan-1	34.80	240.9 -> 205.9	276.7 -> 240.9
Endosulfan-2	38.65	206.9 -> 172.0	241.0 -> 206.0
Endosulfan-sulphate	40.74	271.9 -> 236.9	273.8 -> 238.9
Endrin	34.82	262.9 -> 193.0	263.0 -> 190.9
Ethalfluralin	19.78	275.9 -> 202.1	315.9 -> 275.9
Etofenprox	51.02	163.0 -> 107.0	163.0 -> 135.0
Etoxazole	42.50	141.0 -> 113.0	141.0 -> 63.0
Etridiazole	15.94	211.1 -> 183.0	211.0 -> 140.0
Etrimfos	25.03	181.0 -> 153.1	292.1 -> 181.0
Fenitrothion	29.27	277.0 -> 260.0	277.1 -> 109.0
Fenpropathrin	42.67	97.1 -> 55.1	181.0 -> 151.9
Fenson	31.85	268.0 -> 77.0	268.0 -> 141.0
Fipronil	31.39	367.0 -> 213.0	369.0 -> 215.0
Flonicamid	20.12	146.0 -> 126.0	174.0 -> 69.0
Flucythrinate	50.16	156.9 -> 107.1	198.9 -> 157.0
Fluquinconazole	48.80	340.0 -> 298.0	340.0 -> 108.1
Fluvalinate	52.52	250.0 -> 55.0	250.0 -> 200.0
Folpet	17.69	104.0 -> 76.0	147.0 -> 103.0

Formothion	27.13	126.0 -> 93.0	224.0 -> 125.0
Furalaxyl	32.70	242.0 -> 95.0	301.0 -> 225.0
gamma-BHC	25.08	216.9 -> 181.0	181.0 -> 145.0
Heptachlor	28.25	271.7 -> 236.9	273.7 -> 238.9
Heptachlor epoxide-cis	32.64	352.8 -> 262.9	354.8 -> 264.9
Heptachlor epoxide-trans	32.93	183.0 -> 155.0	182.9 -> 118.9
Hexachlorobenzene	23.07	283.8 -> 248.8	283.8 -> 213.9
Hexazinone	41.10	171.0 -> 71.1	171.0 -> 85.1
Imazalil	35.31	173.0 -> 145.0	215.0 -> 173.0
iodofenphos	35.24	377.0 -> 362.0	379.0 -> 364.0
Isobenzan	30.94	310.8 -> 274.8	310.8 -> 240.9
Isobenzan	30.88	310.8 -> 274.8	310.8 -> 240.9
Isocarbophos	30.98	121.0 -> 65.0	136.0 -> 108.0
Isodrin	36.46	262.9 -> 192.9	262.9 -> 227.9
Isofenphos	31.60	212.9 -> 121.1	212.9 -> 185.1
Isofenphos-methyl	30.99	199.0 -> 121.0	199.0 -> 65.0
Lindane	22.91	180.8 -> 145.0	218.8 -> 183.0
Mecarbam	32.15	130.9 -> 86.0	130.9 -> 74.0
Methiocarb	18.70	168.0 -> 153.0	153.0 -> 45.0
Methoxychlor	43.19	227.0 -> 212.0	227.0 -> 169.0
Metrafenone	46.37	393.0 -> 363.0	393.0 -> 335.0
Naled	11.88	185.0 -> 93.0	185.0 -> 109.0
Naphtalene	11.63	128.0 -> 102.0	128.0 -> 127.0
Nitrofen	37.72	202.0 -> 139.1	283.0 -> 162.0
Ofurace	39.84	232.0 -> 158.0	232.0 -> 186.0
Oxyfluorfen	35.51	252.0 -> 196.0	252.0 -> 146.0
Parathion-ethyl	30.51	291.1 -> 109.0	138.9 -> 109.0
Parathion-methyl	28.34	124.9 -> 47.0	232.9 -> 109.0
Pentachloroanisole	23.09	265.0 -> 237.0	280.0 -> 265.0
Pentachlorobenzene	18.02	249.9 -> 212.9	249.9 -> 179.9
Permethrin	47.83	163.0 -> 127.0	183.1 -> 168.1
Phosphamidon	26.79	264.1 -> 127.0	127.0 -> 95.0
Pirimicarb	25.84	238.0 -> 166.2	166.0 -> 55.1
Pirimicarb desmethyl	26.92	152.0 -> 96.0	152.0 -> 42.0
Pirimiphos-ethyl	30.28	318.1 -> 166.1	318.1 -> 182.1
Procymidone	33.09	283.1 -> 95.9	283.0 -> 67.0
Profluralin	22.99	317.9 -> 199.0	317.9 -> 54.8
Prometryn	28.25	226.0 -> 184.2	241.0 -> 184.0
Propachlor	19.86	120.0 -> 92.0	176.1 -> 120.0
Pyrazophos	45.71	232.0 -> 204.1	221.0 -> 148.0
Pyridaphenthion	42.31	340.0 -> 199.0	125.0 -> 97.0
Pyriproxyfen	45.02	136.1 -> 78.0	136.1 -> 96.0
Quintozene	24.33	295.0 -> 237.0	293.0 -> 235.0
Rimsulfuron	43.86	231.0 -> 216.0	231.0 -> 188.0
Simazine	23.94	201.1 -> 173.1	201.0 -> 186.0
Spiromesifen	41.00	272.0 -> 254.0	272.0 -> 109.0
Sulprofos	38.87	156.0 -> 141.0	156.0 -> 97.0

Tebupirimfos	25.04	233.9 -> 110.1	260.8 -> 137.2
Tecnazene	19.74	260.9 -> 203.0	214.9 -> 179.0
Tefluthrin	24.14	177.1 -> 127.1	177.1 -> 87.0
Telodrin	30.95	311.0 -> 275.0	311.0 -> 241.0
Tetrachlorvinphos	33.81	328.9 -> 109.0	330.9 -> 109.0
Tetradifon	44.90	158.9 -> 131.0	226.9 -> 199.0
Thiabendazole	34.81	201.0 -> 174.0	174.0 -> 130.0
Thiometon	32.55	125.0 -> 47.0	125.0 -> 79.0
Toclofos-methyl	27.92	265.0 -> 250.0	265.0 -> 220.0
Trichlorfon	24.41	145.0 -> 109.0	256.9 -> 109.0
Trichloronat	30.62	296.8 -> 268.9	298.8 -> 270.9
Triflumizole	32.52	206.1 -> 179.0	179.0 -> 144.0
Trifluralin	20.06	305.9 -> 264.0	264.0 -> 206.0
Vinclozolin	27.47	197.9 -> 145.0	198.0 -> 198.0

1.3 LC-MS analysis

LC-MS/MS analysis was performed using a triple quadrupole mass spectrometer (Agilent G6420A) coupled to an Agilent UHPLC system comprising a degasser (G4225A), a binary pump (G1312B), an autosampler (G7129A), and a thermostat column compartment (G1316A). Chromatographic separation was carried out on a Zorbax Eclipse Plus C18 column (2.1 × 50 mm, 1.8 µm) maintained at 40 °C. The mobile phases consisted of (A) water with 0.1% formic acid and (B) methanol with 0.1% formic acid. The chromatographic gradient was as follows: 15% B held for 0.45 min, increased from 15% to 55% B over 1.15 min, then from 55% to 80% B over 9 min, and held at 80% B for 1 min. The flow rate was maintained at 0.3 mL/min, and the injection volume was 5 µL.
Mass spectrometric detection was performed in electrospray ionization (ESI) positive mode. Source parameters were as follows: gas temperature 320 °C, gas flow 12 L/min, nebulizer pressure 60 psi, and capillary voltage 3000 V. Data were acquired in multiple reaction monitoring (MRM) mode.

Table S3. Liquid chromatography tandem mass spectrometry (LC-MS) parameters of pesticides.

Name	*Retention time (min)*	**Quantifying ions (*m/z*)**	**Qualifying ions (*m/z*)**
2,4-dichlorobenzamide	2,93	190.0 -> 172.9	190.0 -> 109.0
Acetamiprid	5,49	223.1 -> 126.0	223.1 -> 56.0
Alachlor	9,81	270.1 -> 238.1	270.1 -> 162.1
Aldicarb	6,07	116.1 -> 89.0	116.1 -> 70.0
Aldicarb-Sulfone	1,88	240.0 -> 86.0	240.0 -> 148.0
metryn	7,71	228.1 -> 186.1	228.1 -> 96.1
Aminocarb	0,98	209.1 -> 137.1	209.1 -> 152.1
Ancymidol	6,78	257.0 -> 81.0	257.0 -> 135.0
Atrazine	7,44	216.1 -> 174.1	216.1 -> 104.0
Azaconazole	7,74	300.0 -> 159.0	302.0 -> 161.0
Azamethiphos	6,54	325.0 -> 183.0	325.0 -> 139.0
Azinphos-ethyl	9,63	346.0 -> 132.1	346.0 -> 233.0
Azinphos-methyl	8,09	318.0 -> 125.0	318.0 -> 77.1
Azoxystrobin	8,55	404.1 -> 372.1	404.1 -> 344.1
Benalaxyl	11,12	326.2 -> 148.1	326.2 -> 294.0
Bendiocarb	6,65	224.1 -> 109.0	224.1 -> 167.1

Benodanil	7,34	324.0 -> 231.0	324.0 -> 203.0
Benoxacor	8,05	260.0 -> 149.0	262.0 -> 149.0
Bensulfuron-methyl	8,16	411.1 -> 149.1	411.1 -> 182.0
Benzovindiflupyr	11,06	398.0 -> 342.0	400.0 -> 380.0
Benzoximate	11,78	364.1 -> 199.0	364.1 -> 105.0
Benzoylprop-Ethyl	11,40	366.1 -> 105.0	366.1 -> 320.0
Bifenazate	9,50	301.2 -> 198.0	301.2 -> 170.0
Bitertanol	11,71	338.2 -> 99.0	338.2 -> 70.0
Boscalid	8,83	343.0 -> 307.1	343.0 -> 271.0
Brodifacoum	15,76	523.0 -> 335.0	523.0 -> 178.0
Bromoxynil	6,99	276.0 -> 80.9	276.0 -> 79.0
Bromuconazole	10,36	378.0 -> 159.0	376.0 -> 70.0
Bupirimate	9,77	317.2 -> 166.1	317.2 -> 108.0
Buprofezin	13,40	306.2 -> 201.1	306.2 -> 116.1
Butachlor	13,79	312.2 -> 238.0	312.2 -> 162.0
Butafenacil	9,87	492.1 -> 330.8	492.1 -> 348.8
Buturon	7,59	237.0 -> 84.0	237.0 -> 126.0
Cadusafos	12,12	271.1 -> 159.0	271.1 -> 131.0
Carbaryl	6,89	202.1 -> 145.1	202.1 -> 127.0
Carbetamide	6,34	237.1 -> 118.0	237.1 -> 192.1
Carbofuran	7,37	221.7 -> 123.0	221.7 -> 165.0
Carbofuran-3-hydroxy	5,50	255.1 -> 163.1	255.1 -> 220.1
Carfentrazone-ethyl	10,77	412.0 -> 346.0	412.0 -> 366.0
Carpropamide	10,95	334.1 -> 139.0	334.1 -> 196.0
Chlorantraniliprole	8,13	484.0 -> 453.0	482.0 -> 284.0
Chlorbromuron	8,56	293.0 -> 182.0	295.0 -> 206.0
Chlorfenvinphos	11,32	359.0 -> 155.1	359.0 -> 99.0
Chloridazon	5,41	222.0 -> 104.1	222.0 -> 92.1
Chlorotoluron	7,21	213.1 -> 72.0	213.1 -> 140.0
Chloroxuron	9,31	291.1 -> 72.0	291.1 -> 218.0
Chlorpropham	9,01	214.1 -> 172.0	214.1 -> 154.0
Chlorpyrifos-methyl	11,83	321.9 -> 125.0	321.9 -> 289.9
Clethodim	12,88	360.0 -> 163.8	360.1 -> 164.1
Clodinafop-propargyl	10,74	350.1 -> 265.9	350.1 -> 90.9
Clofentezine	11,41	303.0 -> 138.0	303.0 -> 102.0
Clomazone	8,10	240.1 -> 125.0	240.1 -> 89.0
Cloquinocet-mexyl	13,77	336.1 -> 237.9	336.1 -> 178.9
Clothianidin	4,91	250.0 -> 169.0	250.0 -> 132.0
Coumaphos	11,15	363.0 -> 227.0	363.0 -> 307.0
Crotoxyphos	8,85	332.0 -> 193.0	332.0 -> 211.0
Cyanazine	6,44	241.1 -> 214.1	241.1 -> 104.0
Cyazofamid	9,96	325.1 -> 108.0	327.0 -> 108.0
Cycloate	11,90	216.1 -> 83.0	216.1 -> 154.1
Cycloxydim	12,54	326.2 -> 280.1	326.2 -> 180.0
Cycluron	7,76	199.2 -> 72.0	199.2 -> 89.0
Cyflufenamid	11,96	413.1 -> 295.0	413.1 -> 241.0
Cyhalothrin	15,41	467.1 -> 225.0	467.1 -> 450.0

Cymoxanil	5,68	199.1 -> 128.1	199.1 -> 111.0
Cypermethrin	15,48	433.3 -> 191.0	435.3 -> 193.0
Cyproconazole	8,99	292.1 -> 125.0	294.0 -> 70.0
Cyprodinil	9,86	226.1 -> 93.0	226.1 -> 108.0
Dazomet	1,89	163.0 -> 90.0	163.0 -> 119.9
Demeton-S-Methyl	6,75	231.0 -> 89.0	231.0 -> 61.0
Demeton-S-Methylsulfone	3,21	263.0 -> 109.0	263.0 -> 169.0
Diazinon	11,14	305.0 -> 169.0	305.0 -> 97.0
Diclobutrazol	10,67	328.1 -> 70.0	330.0 -> 70.0
Diclofop-Methyl	13,42	358.0 -> 281.0	358.0 -> 120.0
Dicrotophos	5,02	238.1 -> 127.0	238.1 -> 112.1
Diethofencarb	8,49	268.2 -> 226.1	268.2 -> 152.0
Difenoconazole	12,21	406.1 -> 251.0	406.1 -> 337.0
Diflubenzuron	10,13	311.0 -> 158.0	311.0 -> 113.0
Diflufenican	12,60	395.1 -> 246.0	334.2 -> 125.1
Dimefuron	8,21	339.1 -> 72.0	339.1 -> 167.0
Dimethenamid (Somma)	8,65	276.1 -> 244.1	278.0 -> 246.0
Dimethoate	5,25	230.0 -> 199.0	230.0 -> 125.0
Dimethomorph	9,07	388.1 -> 301.1	388.1 -> 165.0
Dimoxystrobin	10,56	327.2 -> 116.0	327.2 -> 205.0
Diniconazole	11,80	326.1 -> 70.0	326.1 -> 159.0
Dinotefuran	1,55	203.1 -> 129.0	203.1 -> 114.0
Dioxacarb	6,65	224.1 -> 123.1	224.1 -> 167.1
Diphenamid	7,93	240.1 -> 134.0	240.1 -> 91.0
Dithiopyr	12,89	402.0 -> 354.0	402.1 -> 272.0
Diuron	7,60	233.0 -> 72.0	235.0 -> 72.0
Dodemorph	7,81	282.3 -> 116.1	282.3 -> 98.1
Dodine	11,39	228.3 -> 56.9	228.3 -> 59.9
EPN	12,17	324.1 -> 157.0	324.1 -> 296.0
Epoxiconazole	9,96	330.1 -> 121.0	330.1 -> 100.9
Esprocarb	13,41	266.2 -> 91.0	266.2 -> 71.0
Etaconazole	9,86	328.1 -> 159.0	330.0 -> 161.0
Ethiofencarb sulfone	4,86	258.1 -> 107.1	258.1 -> 201.1
Ethiofencarb sulfoxide	5,42	242.1 -> 107.1	242.1 -> 185.1
Ethiofencarb	7,09	226.1 -> 107.1	226.1 -> 164.1
Ethion	14,02	385.0 -> 199.0	385.0 -> 171.0
Ethiprole	8,76	397.0 -> 351.3	397.0 -> 255.4
Ethirimol	6,15	210.2 -> 98.1	210.2 -> 140.1
Ethofumesate	8,49	304.1 -> 287.1	304.1 -> 241.1
Ethoprophos	9,83	243.1 -> 97.0	243.1 -> 131.0
Ethoxyquin	7,98	218.2 -> 174.1	218.2 -> 148.0
Etofenprox	16,30	394.2 -> 177.1	394.2 -> 359.0
Etoxazole	14,93	360.2 -> 141.0	360.2 -> 113.0
Famoxadone	11,43	392.2 -> 331.1	392.2 -> 238.1
Fenamidone	8,69	312.1 -> 92.1	312.1 -> 236.1
Fenamiphos	10,36	304.1 -> 217.0	304.1 -> 234.0
Fenarimol	9,68	331.0 -> 81.0	331.0 -> 268.0

Fenazaquin	15,42	307.2 -> 57.2	307.2 -> 161.1
Fenbuconazol	10,18	337.1 -> 70.0	337.1 -> 125.0
Fenhexamid	9,71	302.1 -> 97.1	302.1 -> 55.1
Fenobucarb	8,33	208.1 -> 95.0	208.1 -> 152.0
Fenoxanil	10,32	329.1 -> 302.0	329.1 -> 189.0
Fenoxycarb	10,42	302.1 -> 88.0	302.1 -> 116.1
Fenpropimorph	8,37	304.3 -> 147.1	304.3 -> 130.0
Fenpyroximate	15,23	422.2 -> 366.1	422.2 -> 135.0
Fensulfothion	7,67	309.0 -> 173.0	309.0 -> 253.0
Fenthion	10,75	279.0 -> 169.1	279.0 -> 247.0
Fenthion-Sulfoxide	6,88	295.0 -> 280.0	295.0 -> 109.0
Florasulam	6,08	360.0 -> 129.0	360.0 -> 192.0
Fluazifop-P-butyl	13,63	384.1 -> 282.1	384.1 -> 328.1
Fludioxonil	8,66	266.1 -> 229.1	266.1 -> 158.1
Flufenacet	9,87	364.1 -> 152.1	364.1 -> 194.1
Fluometuron	7,13	233.1 -> 72.0	233.1 -> 160.0
Fluopicolide	9,10	383.0 -> 173.0	383.0 -> 145.0
Fluopyram	9,66	397.0 -> 173.0	397.0 -> 208.0
Fluorochloridone	9,33	312.0 -> 89.0	312.0 -> 292.0
Fluoxastrobin	9,78	459.1 -> 427.1	459.1 -> 188.0
Fluquinconazole	9,55	376.0 -> 307.0	376.0 -> 349.0
Fluroxypyr-meptyl	14,76	367.0 -> 181.0	367.0 -> 255.0
Flusilazole	10,39	316.1 -> 247.1	316.1 -> 165.0
Flutriafol	7,50	302.1 -> 70.0	302.1 -> 123.0
Forchlorfenuron	7,62	248.1 -> 129.1	248.1 -> 155.1
Fosthiazate	7,22	284.1 -> 104.0	284.1 -> 228.0
Fuberidazole	5,06	185.1 -> 157.1	185.1 -> 156.0
Furathiocarb	13,46	383.2 -> 195.1	383.2 -> 252.1
Griseofulvin	7,48	353.0 -> 69.0	353.0 -> 165.0
Haloxyfop-methyl	12,38	376.1 -> 316.0	376.1 -> 288.0
Heptenophos	7,76	251.0 -> 127.0	251.0 -> 125.0
Hexaconazole	11,29	314.1 -> 70.0	316.0 -> 70.0
Hexazinone	6,69	253.2 -> 171.1	253.2 -> 71.1
Hexythiazox	14,28	353.1 -> 168.1	353.1 -> 228.0
Imidacloprid	5,01	256.1 -> 209.0	256.1 -> 175.0
Indoxacarb	12,77	528.1 -> 150.0	528.1 -> 203.0
Ioxynil	7,66	369.8 -> 126.9	369.8 -> 214.9
Ipconazole	12,61	334.2 -> 70.1	334.2 -> 125.1
Iprovalicarb	9,69	321.2 -> 119.1	321.2 -> 203.1
Isoprocarb	7,42	211.0 -> 95.0	211.0 -> 137.1
Isoprothiolane	9,02	291.1 -> 189.0	291.1 -> 231.0
Isoproturon	7,57	207.2 -> 72.0	207.2 -> 165.1
Isoxaben	9,05	333.2 -> 165.0	333.2 -> 150.0
Isoxadifen-ethyl	10,56	296.1 -> 232.0	296.1 -> 263.0
Kresoxim-methyl	10,66	314.1 -> 267.0	314.1 -> 116.0
Lenacil	7,50	235.1 -> 153.1	235.1 -> 136.1
Linuron	8,29	249.0 -> 160.0	249.0 -> 182.0

Lufenuron	14,44	511.0 -> 141.0	511.0 -> 158.0
Malaoxon	6,81	315.1 -> 99.0	315.1 -> 127.0
Malathion	9,05	331.0 -> 126.9	331.0 -> 99.0
Mandipropamid	9,07	412.1 -> 328.0	412.1 -> 356.0
Mecarbam	9,74	330.1 -> 97.0	330.1 -> 199.0
Mefenacet	9,35	299.1 -> 120.0	299.1 -> 148.0
Mefenpyr-Diethyl	11,40	373.1 -> 327.0	373.1 -> 160.0
Mepanipyrim	9,39	224.1 -> 77.0	224.1 -> 106.0
Mepronil	9,08	270.0 -> 119.1	270.2 -> 228.0
Mesosulfuron-methyl	7,44	504.1 -> 182.1	504.1 -> 83.1
Metalaxyl	7,70	280.2 -> 220.1	280.2 -> 192.0
Metamitron	5,16	203.1 -> 175.1	203.1 -> 104.1
Metazachlor	7,57	278.1 -> 134.1	278.1 -> 210.1
Metconazole	11,46	320.2 -> 70.0	322.0 -> 70.0
Methabenzthiazuron	7,37	222.1 -> 165.1	222.1 -> 150.0
Methamidophos	0,94	142.0 -> 94.0	142.0 -> 125.0
Methiocarb Sulfoxide	5,42	242.1 -> 185.0	242.1 -> 122.0
Methiocarb	8,50	226.1 -> 121.1	226.1 -> 169.1
Methomyl	2,61	163.1 -> 88.0	163.1 -> 106.0
Methoprotryne	7,88	272.2 -> 198.1	272.2 -> 170.1
Metobromuron	7,28	259.0 -> 170.0	259.0 -> 148.1
Metolachlor	10,00	284.1 -> 252.1	286.0 -> 254.0
Metolcarb	6,28	166.1 -> 109.1	166.1 -> 91.0
Metoxuron	6,12	229.1 -> 72.0	229.1 -> 156.0
Metrafenone	11,75	409.0 -> 227.0	409.0 -> 209.0
Metribuzin	6,56	215.1 -> 84.1	215.1 -> 49.1
Metsulfuron-methyl	6,71	382.1 -> 167.1	382.1 -> 141.0
Mevinphos (E+Z)	5,49	225.1 -> 127.0	225.1 -> 193.0
MKG-264	12,04	276.0 -> 98.0	276.0 -> 210.0
Monocrotophos	4,06	224.1 -> 127.1	224.1 -> 98.1
Monolinuron	6,99	215.1 -> 126.0	215.1 -> 148.1
Monuron	6,40	199.0 -> 72.1	199.0 -> 125.9
Myclobutanil	9,25	289.1 -> 70.0	289.1 -> 125.0
Napropamide	9,85	272.2 -> 129.1	272.2 -> 171.1
Neburon	10,43	275.1 -> 57.0	275.1 -> 88.1
Nuarimol	8,52	315.1 -> 81.0	315.1 -> 252.1
Ofurace	6,74	282.1 -> 160.1	282.1 -> 254.1
Omethoate	0,64	214.0 -> 125.0	214.0 -> 183.0
Oxadiazon	13,84	345.1 -> 185.1	345.1 -> 220.1
Oxadixyl	6,34	279.1 -> 219.1	279.1 -> 133.0
Oxamyl	2,19	237.1 -> 72.0	237.1 -> 90.1
Oxamyloxim	1,89	163.1 -> 72.1	163.1 -> 90.0
Oxycarboxin	5,71	268.1 -> 175.0	268.1 -> 147.0
Paclobutrazol	8,99	294.1 -> 70.0	296.0 -> 70.0
Paraoxon-methyl	6,24	248.0 -> 90.0	248.0 -> 202.0
Parathion	10,42	292.0 -> 264.0	292.0 -> 94.1
Parathion-methyl	8,32	264.0 -> 125.0	264.0 -> 232.0

Penconazole	10,68	284.1 -> 70.0	284.1 -> 159.0
Pencycuron	11,95	329.1 -> 125.0	329.1 -> 218.0
Pendimethanil	16,33	282.1 -> 212.1	282.1 -> 194.1
Phenmedipham	8,08	301.1 -> 136.1	301.1 -> 168.1
Phenothrin	16,17	351.0 -> 183.0	351.0 -> 168.0
Phenthoate	10,57	321.0 -> 247.0	321.0 -> 163.0
Phosalone	11,62	368.0 -> 182.0	368.0 -> 111.0
Phosmet	6,28	318.0 -> 160.0	318.0 -> 77.0
Phosmet oxon	8,08	302.0 -> 160.2	302.0 -> 133.2
Phoxim	11,46	299.1 -> 77.0	299.1 -> 129.0
Picoxystrobin	10,55	368.1 -> 145.1	368.1 -> 205.1
Piperonyl butoxide	13,84	356.2 -> 177.1	356.2 -> 119.0
Piperophos	12,44	354.1 -> 171.0	354.1 -> 255.0
Pirimicarb	6,39	239.2 -> 72.0	239.2 -> 182.1
Pirimicarb, Desmethyl-	5,14	225.0 -> 72.0	225.0 -> 168.0
Pirimiphos-methyl	11,43	306.1 -> 108.1	306.1 -> 164.1
Pirimisulfuron-Methyl	9,27	467.0 -> 226.0	467.0 -> 176.0
Prochloraz	11,25	376.0 -> 308.0	376.0 -> 266.0
Profenofos	12,99	372.9 -> 302.9	372.9 -> 344.9
Promecarb	8,82	208.1 -> 109.1	208.1 -> 151.1
Prometon	7,19	226.2 -> 142.1	226.2 -> 184.1
Prometryn	8,79	242.1 -> 158.1	242.1 -> 200.1
Propamocarb	0,63	189.2 -> 102.0	189.2 -> 74.0
Propanil	8,31	218.0 -> 127.0	218.0 -> 162.0
Propaquizafop	13,61	444.1 -> 100.1	444.1 -> 299.0
Propargite	14,95	368.1 -> 57.1	368.1 -> 175.1
Propazine	8,43	230.1 -> 146.0	230.1 -> 188.1
Propetamphos	9,32	282.1 -> 138.0	282.1 -> 156.0
Propham	7,34	180.1 -> 120.0	180.1 -> 138.1
Propiconazole	11,13	342.1 -> 69.0	342.1 -> 159.0
Propoxur	6,65	210.1 -> 111.0	210.1 -> 168.1
Propyzamide	8,89	256.0 -> 190.0	256.0 -> 173.0
Prosulfocarb	12,70	252.1 -> 91.1	252.1 -> 128.1
Prothiophos	15,64	345.0 -> 241.0	345.0 -> 269.0
Pymetrozine	0,63	218.1 -> 105.0	218.1 -> 78.0
Pyraclostrobin	11,57	388.1 -> 163.0	388.1 -> 194.1
Pyrethrines (Mixture)	15,15	329.0 -> 161.0	329.0 -> 143.0
Pyridaben	15,59	365.2 -> 147.1	365.2 -> 309.1
Pyrifenox	8,85	295.0 -> 93.0	297.0 -> 93.0
Pyrimethanil	7,92	200.1 -> 107.1	200.1 -> 183.0
Quinalphos	10,44	299.1 -> 163.0	299.1 -> 147.1
Quinoxyfen	13,83	308.0 -> 197.0	308.0 -> 272.0
Quizalofop-ethyl	13,06	373.1 -> 299.1	373.1 -> 271.1
Resmethrin	15,86	339.2 -> 171.0	356.2 -> 128.1
Rotenone	10,33	395.2 -> 213.1	395.2 -> 192.1
Sethoxydim	13,39	328.2 -> 178.1	328.2 -> 282.2
Simeconazole	9,80	294.1 -> 70.1	294.1 -> 135.1

Simetryn	6,82	214.1 -> 124.1	214.1 -> 144.1
Spinosad (Spinosyn A)	12,07	732.5 -> 142.0	732.5 -> 98.0
Spinosad (Spinosyn D)	12,92	746.5 -> 142.0	746.5 -> 98.4
Spirodiclofen	15,28	411.1 -> 71.2	411.1 -> 313.0
Spirotetramat	9,84	374.2 -> 302.2	374.2 -> 216.0
Spiroxamine	8,70	298.3 -> 144.1	298.3 -> 100.1
Sulfotep	10,81	323.0 -> 171.0	323.0 -> 143.0
tau-Fluvalinate	15,93	503.1 -> 180.9	503.0 -> 208.0
Tebuconazole	10,88	308.2 -> 70.0	310.0 -> 70.0
Tebufenozide	10,54	353.2 -> 133.1	353.2 -> 297.2
Tebufenpyrad	13,57	334.2 -> 145.1	334.2 -> 117.0
Tebuthiuron	6,83	229.1 -> 172.1	229.1 -> 116.0
Teflubenzuron	13,51	381.0 -> 158.0	381.0 -> 141.0
Tepraloxydim	9,60	342.2 -> 250.1	342.2 -> 166.1
Terbumeton	7,31	226.2 -> 170.1	226.2 -> 114.1
Terbuthylazine	8,65	230.0 -> 174.0	232.0 -> 176.0
Terbutryn	8,92	242.1 -> 186.1	242.1 -> 96.0
Tetraconazole	9,90	372.0 -> 159.0	372.0 -> 70.0
Tetramethrin	13,44	332.2 -> 164.0	332.2 -> 135.0
Thiabendazole	4,53	202.0 -> 175.0	202.0 -> 131.1
Thiacloprid	5,83	253.0 -> 126.0	255.0 -> 128.0
Thidiazuron	6,58	221.1 -> 102.0	221.1 -> 127.8
Thifensulfuron-methyl	6,57	388.0 -> 167.1	388.0 -> 205.0
Thiobencarb	11,65	258.1 -> 125.1	260.0 -> 127.0
Thiodicarb	7,17	355.1 -> 88.0	355.1 -> 108.0
Thiophanate-Methyl	6,61	343.1 -> 151.0	343.1 -> 93.0
Tralkoxydym	14,25	330.2 -> 138.0	330.0 -> 96.0
Triadimefon	9,21	294.1 -> 69.0	294.1 -> 197.0
Triadimenol	9,50	296.1 -> 70.0	298.0 -> 70.0
Triazophos	9,43	314.1 -> 162.1	314.1 -> 119.0
Trichlorfon	5,20	256.9 -> 109.0	256.9 -> 221.0
Tricyclazole	6,02	190.0 -> 136.0	190.0 -> 163.0
Trietazine	9,51	230.1 -> 99.0	230.1 -> 202.0
Trifloxystrobin	12,70	409.1 -> 186.1	409.1 -> 206.1
Triflumizole	12,49	346.1 -> 278.1	346.1 -> 73.1
Triflumuron	11,69	359.0 -> 156.0	359.0 -> 139.0
Tritosulfuron	8,19	444.4 -> 192.9	444.4 -> 135.9
Vamidothion	5,53	288.1 -> 146.1	288.1 -> 118.0
Zoxamide	11,00	336.0 -> 187.0	336.0 -> 159.0

Figure S1: Ascorbic acid calibration curve (DPPH test).

Ascorbic acid solution	Concentration (mM)
C0	0
C1	1
C2	0.8
C3	0.5
C4	0.2
C5	0.1

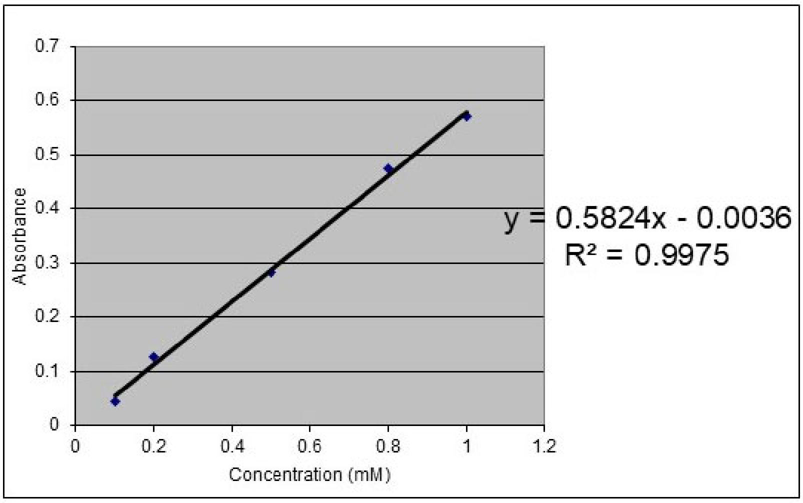

Supplement: Supplementary file 1 [file foods-14-03269-s001.zip › foods-3831107-supplementary.pdf]
